# Neuropsychiatric Lupus: A Challenging Journey of a Patient With Pulmonary Tuberculosis

**DOI:** 10.7759/cureus.16018

**Published:** 2021-06-29

**Authors:** Ammar Farook Chapra, Fadi Khir, Ans Alamami, Khaled M Salem, Alhady Yusof

**Affiliations:** 1 Internal Medicine, Hamad Medical Corporation, Doha, QAT; 2 Internal Medicine Residency Program, Medical Education, Hamad Medical Corporation, Doha, QAT; 3 Critical Care, Hamad Medical Corporation, Doha, QAT; 4 Neuroradiology Section, Neuroscience Institute, Hamad Medical Corporation, Doha, QAT

**Keywords:** systemic lupus erythematous disease, neuro-critical care, neuropsychiatric sle, active pulmonary tuberculosis, acute encephalitis

## Abstract

Systemic lupus erythematosus (SLE) is a disease that affects multiple systems in the body. Due to its variable manifestations, it can at times pose challenges for physicians to hold SLE as the culprit behind an affected system. This is most true when encountering patients with neuropsychiatric manifestations of SLE. We present a case of a 38-year-old female with known SLE limited to skin involvement and on treatment for active pulmonary tuberculosis (TB), yet otherwise healthy, who presented with acute fever associated with generalized tonic-clonic seizures. She was investigated for meningoencephalitis with a cerebrospinal fluid (CSF) analysis not being fully conclusive and with imaging features suggestive of viral encephalitis. However, despite receiving optimal care for causes of bacterial, viral, and tuberculous meningitis the patient continued to deteriorate and started to develop predominant psychiatric symptoms in the form of confusion and combative behavior requiring pharmacological restraint. Hence a trial of immunosuppressives was given with a presumptive diagnosis of neuropsychiatric lupus with IV methylprednisolone followed by a course of IV cyclophosphamide. However, this treatment proceeded with caution due to the fear of disseminated tuberculosis for which she did not show any sign of in the subsequent weeks. The patient showed modest clinical and radiological improvement and hence the treatment was continued. The case highlights the uncertainty that may precede a diagnosis of neuropsychiatric lupus and the challenges in treating it in patients with active mycobacterial infection.

## Introduction

Systemic lupus erythematosus (SLE) is a multisystem autoimmune disease with a wide range of clinical manifestations as it can affect any organ system in the body. SLE is thought to be multifactorial in origin with environmental factors and genetic predisposition playing a significant role as it has a female predilection with a global ratio of 9:1 [1].

The clinical presentation of SLE depends on which organ system is involved, and to what extent it can surface with varying degrees of severity. For instance, the neurological presentation of lupus can range anywhere from headache, seizure, to intellectual disability and neuropathy. The underlying pathology can be attributed to SLE encephalitis, meningitis, or myelitis. Nevertheless, a common presentation for neuro-SLE is the psychiatric manifestation such as psychosis, depression, anxiety disorder, to name a few [2-3]. 

The diagnosis of SLE is still challenging even with the advancement in technologies and having recognized, detectable autoantibodies, as not all immunological markers will be positive and the changing nature of the presentation depends on the site affected [4].

The treatment for SLE mainly uses immunosuppressive medications, for trying to control the pathological process along with its complications, which at times may pose challenges in the presence of any co-existing infectious process from the time of diagnosis to the acute and long-term maintenance drug determination [5-6]. Here, we are presenting a case of newly diagnosed SLE in a patient with a recent diagnosis of pulmonary tuberculosis (TB) with progressive neurological symptoms.

## Case presentation

A 38-year-old woman from Southeast Asia, recently known to have been diagnosed with TB and SLE, who started on treatment, was brought to our hospital via Emergency Medical Services (EMS) due to fever, recurring seizures, and decreased level of consciousness. 

She had arrived from her home country four months prior and was shortly later admitted to the hospital for an incidental diagnosis of active TB discovered due to chest X-ray findings on pre-employment screening. Her hospital admission, however, was significant for an observation of a baseline mild neutropenia and lymphopenia and upon initiation of first-line anti-TB medications, she had severe worsening of neutropenia and development of neutropenic sepsis attributed to rifampicin. During this admission, she was noticed to have a malar rash and subsequent testing revealed a positive antinuclear antibody (ANA) of 1:1280, positive anti-double stranded DNA (anti-dsDNA) antibody, positive anti-smith antibody (anti-Sm), positive anti-Ro antibody, positive antinuclear ribonucleoprotein (anti-RNP) antibody, and low complement levels. Subsequently, she was diagnosed with SLE and started on hydroxychloroquine. She was discharged on second-line anti-TB medications to avoid isoniazid for its association with drug-induced lupus and rifampicin due to its association with neutropenia which she had previously developed.

However, the patient was found to have stopped her anti-TB regimen and her hydroxychloroquine by herself after discharge which was subsequently resumed a month, later on, follow up. Her subsequent course before the current presentation was also eventful for hospital admission due to severe *Clostridium difficile* sepsis requiring ICU admission. However, the patient was discharged in a healthy condition with no residual neurological deficits. 

A month later, the patient was discovered at home to be unresponsive after an episode of vomitus followed by a tonic seizure aborted with medication. She was also found to have a fever of 39.8 degrees Celsius. She had another focal seizure before arrival to hospital and upon arrival, was observed to have repeated convulsions varying from generalized tonic-clonic seizures to focal seizures lasting minutes aborted with IV lorazepam. No collateral history regarding events before this could be taken. She was neither known to have had any seizures in the past nor was there any history of alcohol or drug abuse. 

**Figure 1:**
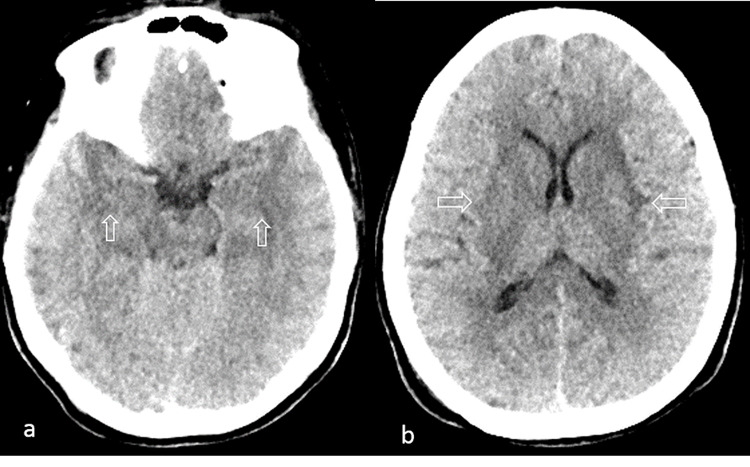
Non-contrast CT scan of the brain. Initial plain CT scan of the brain with axial cuts showing (a) bilateral cerebral temporal and fronto-parietal deep white matter periventricular hypodensities as well as (b) basal ganglia hypodensities (hollow arrows).

Upon initial assessment, she had a Glasgow Coma Scale (GCS) of 8/15 (E2 V2 M4) with hemodynamic stability. She was noted to have bilaterally equal and reactive pupils, three millimeters in size. She had obvious neck stiffness present. The remainder of the exam was unremarkable. Blood investigations showed a mildly elevated neutrophil predominant leukocytosis, with normocytic normochromic anemia. Aside from hypokalemia on initial presentation, all electrolytes were within normal limits. Initial imaging included a CT scan of the brain (Figure 1) which revealed bilateral subcortical and deep white matter hypodensities along with bilateral temporal lobe hypodensities suspicious of viral encephalitis.

She subsequently underwent a lumbar puncture (LP) which revealed a clear and colorless cerebrospinal fluid (CSF). The analysis of CSF is as follows: leukocytes cell count was 27/μL with 60% neutrophils and 1013/μL red blood cells (RBC). Glucose was decreased at 2.06 mmol/L (serum glucose 5.6 mmol/L) with an elevated CSF protein (1.89 g/L normal range 0.15-0.45 g/L). Polymerase chain reaction (PCR) testing for common viruses including HSV-1/HSV-2 were all found to be negative, along with a negative screen for TB PCR and acid-fast bacilli (AFB) smear from CSF. Considering a provisional diagnosis of acute meningoencephalitis of possible viral, bacterial or tuberculous origin, she was started empirically on ceftriaxone, ampicillin, vancomycin, acyclovir, and dexamethasone all intravenously and her home TB medications were resumed. She was initiated on IV levetiracetam for seizures. Following negative Gram stain results, vancomycin was stopped and remaining medications were continued. 

**Figure 2:**
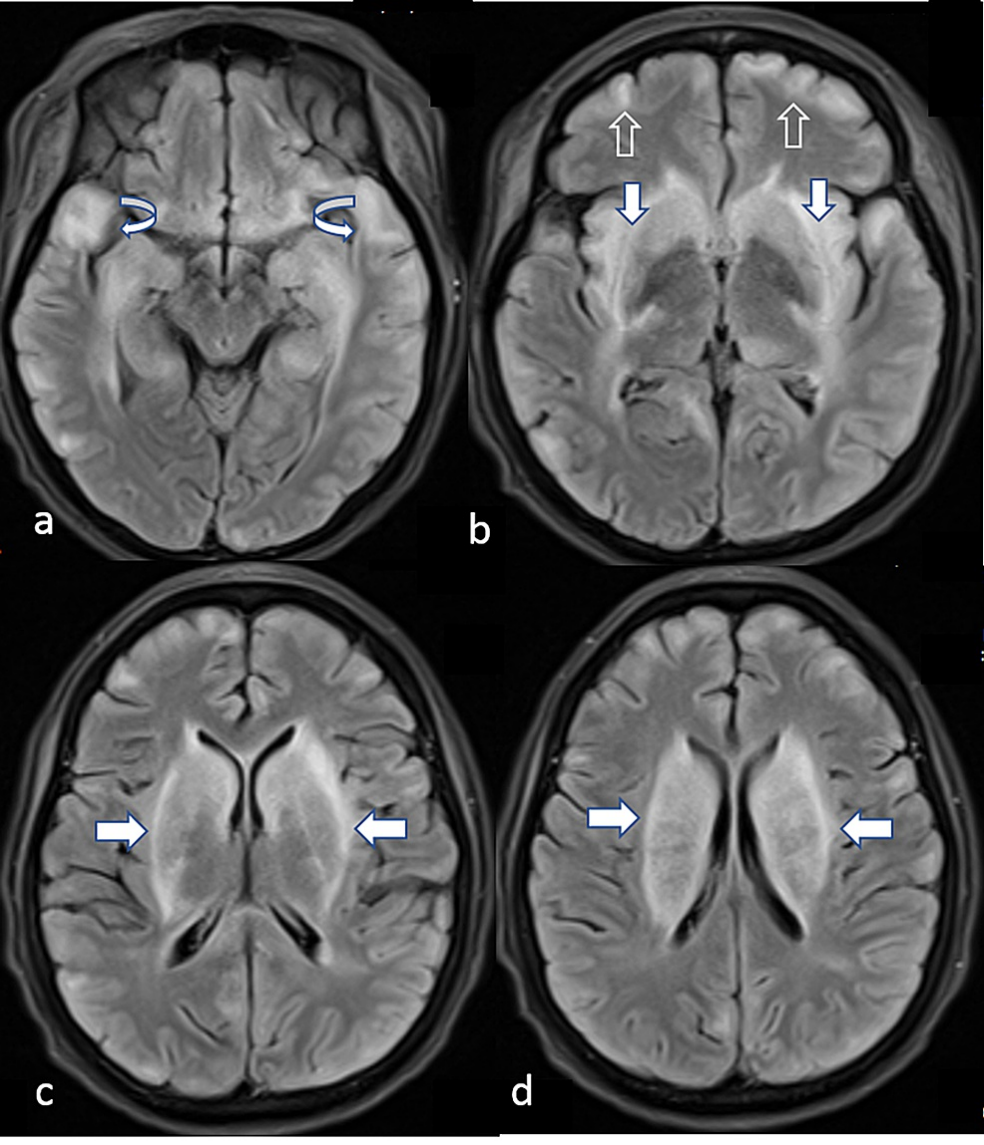
Pre-treatment MRI brain axial FLAIR. (a-d): Pre-treatment MRI brain axial FLAIR showing bilateral rather symmetrical basal ganglia external capsule (horizontal and vertical white arrows) and subcortical white matter (hollow and curved arrows) hyperintense areas in keeping with neuropsychiatric lupus with antibody-mediated striatal encephalitis. FLAIR, fluid-attenuated inversion recovery

MRI of the brain (Figure 2) was done and it showed diffuse areas of diffusion restrictions with no definitive intracranial vascular occlusion.

These findings along with the history of SLE, the low complement levels, and the positive Anti-dsDNA (235 IU/mL reference range for positive test > 15 IU/mL) and anti-SmD (23 U/mL, reference range for positive test > 10 IU/mL) raised the possibility of SLE meningoencephalitis, however, due to the lack of a definitive diagnostic test and the fear of TB dissemination with high doses of immunosuppressant medications; no treatment for SLE other than the hydroxychloroquine was initiated at that time.

Despite the treatment directed toward TB and bacterial meningitis, the patient’s condition continued to worsen and her agitation and confusion were difficult to control despite using high doses of antipsychotics and benzodiazepines.

**Figure 3:**
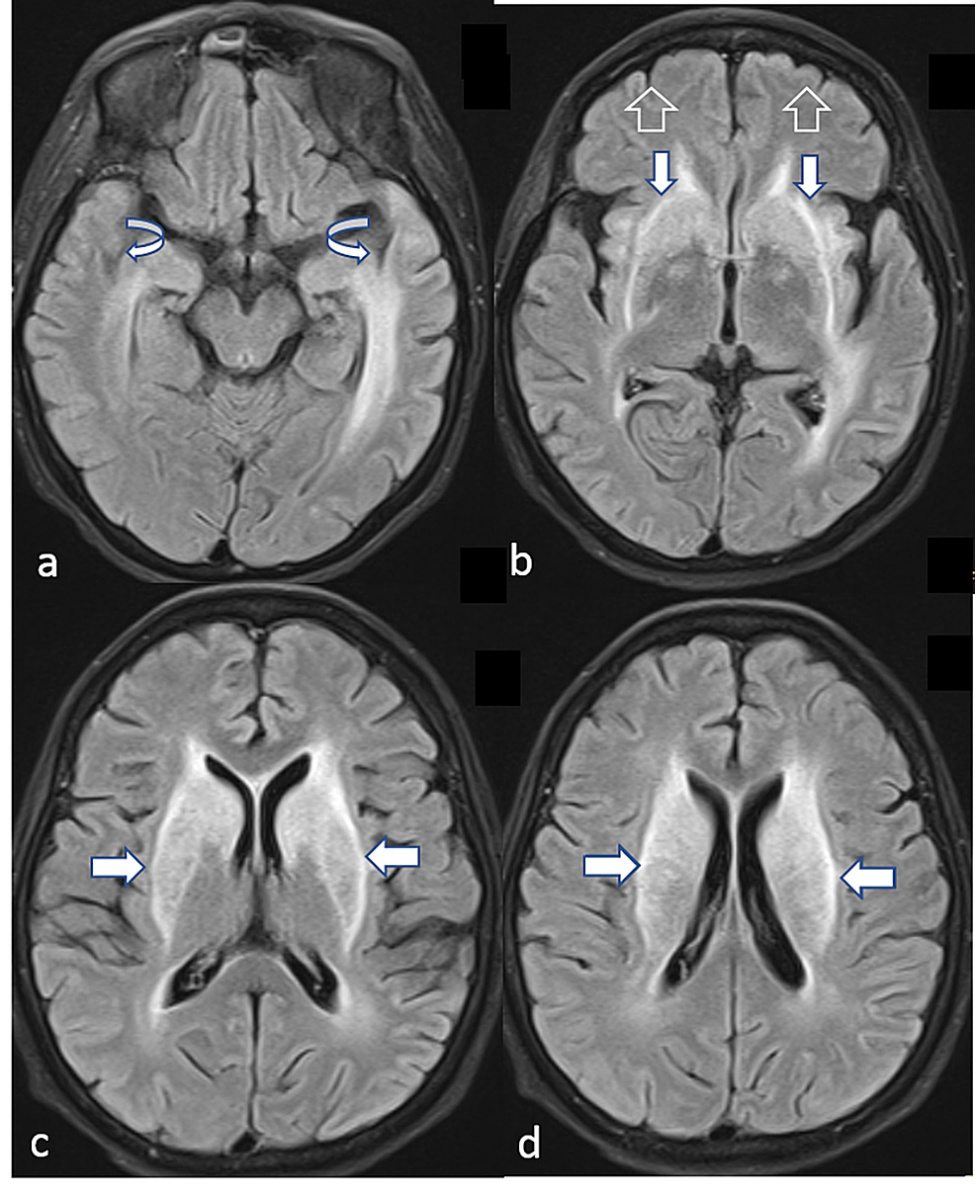
Follow-up MRI axial FLAIR images. (a-d) show mild regression of the hyperintense areas mainly at previously seen subcortical and external capsule regions (denoted by the horizontal, vertical, hollow, and curved arrows). FLAIR, fluid-attenuated inversion recovery

Due to the lack of improvement, it was decided to redirect the treatment towards SLE as a cause of the patient’s neuropsychiatric manifestations and methylprednisolone 500 mg intravenously was given once daily for three days along with the other ongoing treatment over the next few days, while no significant improvement in the clinical state of the patient was noted. Repeat MRI of the brain (Figure 3) showed mild regression of the previously seen diffusion restricted areas and based on these findings it was decided to continue the treatment for SLE with IV cyclophosphamide 500 mg.

Over the two weeks after the first dose of cyclophosphamide the patient remained in a state of confusion, however, she became less agitated, and smaller doses of antipsychotics and benzodiazepines were needed to control her agitation. Hence, the treatment with cyclophosphamide was continued and the patient received four more doses with an interval of two weeks between every other dose along with the prednisolone and the TB treatment. The patient subsequently showed remarkable improvement as she became less confused, started to be able to take care of her basic needs, recognize her name, and follow simple commands.

Fortunately, the patient did not develop any signs or symptoms to suggest dissemination or worsening of her previous TB infection.

## Discussion

The above-mentioned patient’s clinical presentation of decreased level of consciousness with abnormal movements could be explained by different pathologies of which SLE and infectious causes are amongst the top differentials. The initial workup of the CT head, although showed features suggestive of viral infection, would not be able to rule out underlying neuro-psychiatric SLE which could present with the same clinical picture despite a low titer of anti-dsDNA and anti-SmD antibody. The CSF analysis showed a high leukocyte count with predominant neutrophils, high protein, and low glucose, a picture suggestive of ongoing inflammation which is again highly suggestive of infection. At the same time, the CSF viral PCR, bacterial cultures, AFB smear, and PCR were negative yet infectious etiology could not be ruled out.

After the initial assessment, empirical antimicrobial medications were started. However, with regard to the presenting feature of headache, confusion, seizure, visual disturbance, and psychiatric manifestation in terms of psychosis along with the key fact of there being a lack of clinical improvement in the chief findings mentioned above, raised the possibility of CNS manifestation of SLE rather than an infectious etiology. Furthermore, the suspicion was increased considering there was an insignificant response to the already initiated antimicrobial medication although the other systemic manifestations of SLE were not apparent at that time [7].

The diagnosis of neuropsychiatric SLE is challenging as there is no single test with adequate sensitivity or specificity to advocate for an accurate diagnosis. The 2019 European League Against Rheumatism (EULAR)/American College of Rheumatology (ACR) diagnostic criteria (Tables 1-2) has defined lupus as the presence of one clinical criterion at least in addition to 10 points from different clinical and immunological findings.

**Table 1:** EULAR/ACR clinical criteria for diagnosis of SLE. The European League Against Rheumatism (EULAR)/American College of Rheumatology (ACR) clinical criteria for diagnosis of SLE [8]. SLE, systemic lupus erythematosus

Domain	Criteria	Points
Constitutional	Fever	2
Hematologic	Leukopenia	3
	Thrombocytopenia	4
	Autoimmune hemolysis	4
Neuropsychiatric	Delirium	2
	Psychosis	3
	Seizure	5
Mucocutaneous	Non-scarring alopecia	2
	Oral ulcers	2
	Subacute cutaneous or discoid lupus	4
	Acute cutaneous lupus	6
Serosal	Pleural or pericardial effusion	5
	Acute pericarditis	6
Musculoskeletal	Joint involvement	6
Renal	Proteinuria > 0.5 g/24 h	4
	Renal biopsy class II or V lupus nephritis	8
	Renal biopsy class III or IV lupus nephritis	10

**Table 2:** EULAR/ACR immunologic criteria for diagnosis of SLE. The European League Against Rheumatism (EULAR)/American College of Rheumatology (ACR) immunologic criteria for diagnosis of SLE [8]. SLE, systemic lupus erythematosus

Domain	Criteria	Points
Antiphospholipid antibodies	Anti-cardiolipin antibodies or Anti-β2GP1 antibodies or Lupus anticoagulant	2
Complement proteins	Low C3 or low C4	3
	Low C3 and low C4	4
SLE-specific antibodies	Anti-dsDNA antibody or Anti-Smith antibody	6

The combined approach of clinical findings in patients with an established diagnosis of SLE in addition to ruling out other causes mimicking CNS SLE involvement is crucial to reach a precise diagnosis. The role of CSF immunological analysis has been shown to have clinical significance particularly with the utilization of more than one test. In a prospective study by West et al., the patients with SLE hospitalized with the neuropsychiatric disease, 52 patients with neuropsychiatric systemic lupus erythematosus (NPSLE) were categorized by neuropsychiatric presentation (32 diffuse, 10 focal, and 10 complex presentations) and compared to 14 SLE control patients. Each NPSLE patient with a diffuse or complex presentation had abnormal CSF IgG index/oligoclonal bands, elevated CSF antineuronal antibodies, and/or serum anti-ribosomal-P antibodies, yielding a sensitivity of 100%, specificity of 86%, and positive predictive value (PPV) of 95% for this combination of tests. Nine out of 10 patients with focal presentations and all with the complex disease had evidence of vasculitis/livedo reticularis, antiphospholipid antibodies, and/or a cranial MRI with multiple lesions, giving a sensitivity of 95%, specificity of 86%, and a PPV of 90% for this assortment of tests. These tests combined correctly diagnosed all nine SLE patients whose initial diagnosis proved to be incorrect based on the subsequent clinical course [9].

The role of neuro-imaging in neuropsychiatric SLE is of great value and the diagnosis and follow-up [10] as demonstrated in our case were supported by the MRI findings initially and by the clinical and radiological findings’ significant improvement following the initiation of pulse steroid and the cyclophosphamide therapy. The periventricular increased signal (PIS) was a frequent MRI finding inpatient with NPSLE based on a study by Stimmler et al., as MRI abnormality described in 53% of the cases included in a prospective study of 51 patients, though it lacked the specificity of the radiological finding between the NPSLE and SLE without neuropsychiatric manifestations [11].

## Conclusions

Systemic lupus erythematosus is a multi-system disorder that may even present with a neuropsychiatric manifestation such as in our case. Due to the lack of diagnostic criteria for this condition, differentiating between viral meningoencephalitis and neuro-SLE can be challenging leading to delayed initiation of treatment and poorer outcomes. Hence, it is prudent to keep SLE encephalitis in the possibility when a patient known to have SLE presents with fever and altered mental status.
